# Comparative analysis of two phytochrome mutants of tomato (Micro-Tom cv.) reveals specific physiological, biochemical, and molecular responses under chilling stress

**DOI:** 10.1186/s43141-020-00091-1

**Published:** 2020-11-27

**Authors:** Raheel Shahzad, Faraz Ahmed, Zheng Wang, Putri Widyanti Harlina, Elsayed Nishawy, Mohamed Ayaad, Abdul Manan, Mohamed Maher, Mohamed Ewas

**Affiliations:** 1 Department of Biotechnology, Universitas Muhammadiyah Bandung, Bandung, 40614 Indonesia; 2 National Key Laboratory of Crop Genetic Improvement and National Center of Plant Gene Research (Wuhan), Huazhong Agricultural University, Wuhan, 430070 P.R. China; 3 School of Life Sciences, Anhui Agricultural University, Hefei, Anhui P.R. China; 4 Department of Food Technology, Universitas Muhammadiyah Bandung, Bandung, 40614 Indonesia; 5 Department of Plant Genetic Resources, Desert Research Center, Cairo, 11753 Egypt; 6 Egyptian Atomic Energy Nuclear Research Center, Inshas, Egypt; 7 Center for Advance Studies in Vaccinology and Biotechnology, University of Baluchistan, Quetta, 87300 Pakistan; 8 Department of Biochemistry, College of Agriculture, Zagazig University, Zagazig, 44511 Egypt

**Keywords:** Antioxidant enzymes, Chilling stress, Molecular response, Osmolytes, Phytochrome mutants *aur*ea and *high pigment1*, Tomato

## Abstract

**Background:**

Phytochromes are plant photoreceptors that have long been associated with photomorphogenesis in plants; however, more recently, their crucial role in the regulation of variety of abiotic stresses has been explored. Chilling stress is one of the abiotic factors that severely affect growth, development, and productivity of crops. In the present work, we have analyzed and compared physiological, biochemical, and molecular responses in two contrasting phytochrome mutants of tomato, namely *aurea* (*aur*) and *high pigment1* (*hp1*), along with wild-type cultivar Micro-Tom (MT) under chilling stress. In tomato, *aur* is phytochrome-deficient mutant while *hp1* is a phytochrome-sensitive mutant. The genotype-specific physiological, biochemical, and molecular responses under chilling stress in tomato mutants strongly validated phytochrome-mediated regulation of abiotic stress.

**Results:**

Here, we demonstrate that phytochrome-sensitive mutant *hp1* show improved performance compared to phytochrome-deficient mutant *aur* and wild-type MT plants under chilling stress. Interestingly, we noticed significant increase in several photosynthetic-related parameters in *hp1* under chilling stress that include photosynthetic rate, stomatal conductance, stomatal aperture, transpiration rate, chlorophyll a and carotenoids. Whereas most parameters were negatively affected in *aur* and MT except a slight increase in carotenoids in MT plants under chilling stress. Further, we found that PSII quantum efficiency (Fv/Fm), PSII operating efficiency (*Fq*′/*Fm*′), and non-photochemical quenching (NPQ) were all positively regulated in *hp1*, which demonstrate enhanced photosynthetic performance of *hp1* under stress. On the other hand, Fv/Fm and *Fq*′/*Fm*′ were decreased significantly in *aur* and wild-type plants. In addition, NPQ was not affected in MT but declined in *aur* mutant after chilling stress. Noticeably, the transcript analysis show that PHY genes which were previously reported to act as molecular switches in response to several abiotic stresses were mainly induced in *hp1* and repressed in *aur* and MT in response to stress. As expected, we also found reduced levels of malondialdehyde (MDA), enhanced activities of antioxidant enzymes, and higher accumulation of protecting osmolytes (soluble sugars, proline, glycine betaine) which further elaborate the underlying tolerance mechanism of *hp1* genotype under chilling stress.

**Conclusion:**

Our findings clearly demonstrate that phytochrome-sensitive and phytochrome-deficient tomato mutants respond differently under chilling stress thereby regulating physiological, biochemical, and molecular responses and thus establish a strong link between phytochromes and their role in stress tolerance.

**Supplementary Information:**

The online version contains supplementary material available at 10.1186/s43141-020-00091-1.

## Background

Phytochromes are plant pigments that have been found in most plants where it regulates growth, developmental, and adaptive processes. These pigments have unique features to absorb red and far-red light ranging between 660 and 730 nm, which correspond to red and far-red region, respectively [1]. Phytochromes are class of photoreceptors that are important regulator of light-mediated responses known as “photomorphogenesis” in plants [2]. To explore the functional role of phytochromes in plants, its mutants with disruption in biosynthesis or signal transduction could be utilized. These mutants can be helpful in determining phytochrome activity and its correlation with morpho-physiological responses and hence sometimes referred to as “photomorphogenic mutants” in plants. In tomato (*Solanum lycopersicum* L.) plants, the *aur*ea (*aur*) mutant is phytochrome deficient, while the *high pigment1* (*hp1*) mutant is highly sensitive to light-dependent responses [3]. Recently, several studies have shown that phytochromes are closely linked with biotic and abiotic stress responses beside their critical role in photomorphogenesis. For instance, different studies have shown involvement of phytochromes in response to temperature fluctuations, water stress, salt stress, UV-B light, and insect herbivory [4–6]. Given the fact that phytochromes are associated with number of environmental factors, nevertheless, we still lack exact knowledge and underlying regulatory mechanisms of phytochromes in response to different stress factors.

Chilling stress refers to non-freezing low temperature and affects many processes involved during plant growth and development. Many plant species that have been originated from tropical and subtropical regions could be killed or severely injured because they usually failed to tolerate non-freezing chilling temperatures typically below 10 °C [7–9]. Among many other biochemical processes, photosynthesis could be seriously affected by chilling stress which indirectly disturbs the entire physiology of the plants at molecular level. Further, the resulting response of low temperature stress decreases the ability of energy utilization and thus causes the feedback inhibition of photosynthesis in plants [10]. The change in carbohydrate contents under low temperature has also been reported [11]. Moreover, low temperature could significantly affect sugar metabolism and endogenous hormone contents in plants [12]. In general, negative impact of low temperature on MDA, proline, and other low molecular weight molecules has been reported in most plants [13, 14]. In plants, low temperature also induce oxidative damage which is caused by generation of free radicals within the cells such as superoxide anion (O2.^−^), hydroxyl radical (·OH), and hydrogen peroxide (H_2_O_2_). These free radicals are collectively known as reactive oxygen species (ROS) and cause major damage to lipids and proteins in the plant cell [15]. Under normal conditions, ROS production and scavenging processes are in balanced state; however, stress conditions can easily overcome antioxidant defense system which includes both enzymatic and non-enzymatic components [16]. Enzymatic antioxidants contain enzymes such as superoxide dismutase (SOD), catalase (CAT), guaiacol peroxidase (GPX), ascorbate peroxidase (APX), glutathione reductase (GR), and glutathione S-transferases (GST), while non-enzymatic antioxidants comprise of proline, glycine betaine, flavonoids, ascorbic acid, glutathione, carotenoids, and tocopherols [13, 16]. Another phenomenal response which plants show upon exposure to stress conditions is the accumulation of osmolytes. Depending on plant genotype, severity, and type of stress, plants accumulate various osmolytes such as proline, glycine betaine (GB), trehalose, and soluble sugars, which have an active role in maintaining membrane integrity, stabilizing protein, alleviating ionic toxicity, and protecting antioxidant compounds under various stress conditions mainly due to osmotic imbalance [17]. Interestingly, it is evident from previous study which revealed that tomato plants do not accumulate GB under normal situations and hence are more susceptible to chilling stress; however, higher accumulation of GB show enhanced tolerance against chilling temperature in tomato plants [18].

It is interesting to study the link between phytochromes and temperature especially non-freezing chilling temperatures because of the fact that light and temperature affect almost every aspect of plant growth and development. It is obvious that phytochromes actively participate to regulate the expression of cold regulated genes (CORs) in number of plant species and thus play role in cold signaling pathways [19, 20]. To evaluate the role of phytochromes in stress responses, phytochrome-mutant plants were used as experimental material in different studies [5, 21, 22]. Importantly, a thorough evaluation of physiological, biochemical, and other molecular responses in phytochrome-mutant plants under chilling stress is of prime significance and may provide insights into molecular mechanism of phytochrome-mediated responses to abiotic stress.

Tomato (*Solanum lycopersicum* L.) is a cold sensitive plant, and its productivity can be severely affected by chilling temperature at all stages after its plantation. Although several classical reports have utilized phytochrome mutants (mostly single, double or triple phytochrome mutants) to unravel the involvement of specific phytochrome in response to different abiotic stress factors, but the underlying molecular mechanisms are still not known. In tomato, *aur* is a phytochrome-deficient mutant while *hp1* is a phytochrome-sensitive mutant. We employed these two contrasting phytochrome mutants in our research to get insight into molecular mechanisms of phytochrome-mediated responses to chilling stress. Our specific objectives were to unravel physiological, biochemical, and other molecular mechanisms to further deepen our knowledge and establish a strong link between phytochromes and their involvement during chilling stress in tomato plants.

## Methods

### Source of plants

Seeds of wild-type tomato (*Solanum lycopersicum* L.) cultivar Micro-Tom (MT) and its photomorphogenic mutant’s *aur* and *hp1* were obtained from Anhui seed bank, China.

### Plant material and treatment

We have selected uniform and healthy seeds of wild-type tomato (*Solanum lycopersicum* L.) cultivar Micro-Tom (MT) and its photomorphogenic mutants *aur* and *hp1*, which were surface sterilized with 70% ethanol for 5 min. Twenty seeds were then placed onto each petri dish containing Murashige-Skoog (MS) medium and allowed to germinate in an illuminated growth chamber with a 16-h/8-h (light/dark) photoperiod and a photon flux density of 200 μmol m^−2^ s^−1^ at 26 °C for 5 days as described in earlier report with little modifications [23]. After 5 days, the number of seedlings was reduced to half and petri dishes with healthy plants were placed in growth chambers. Two weeks later, the seedlings were shifted to greenhouse in small single-plant pots containing a mixture of sandy loam soil (90%) and manure (10%), having a pH of 7.5. During the experiment period in this study, potted grown tomato plants were placed at a distance of 20 cm to avoid shading and maintained under controlled conditions of temperature (24–27 °C), humidity (60–80%), photoperiod (16-h day/8-h night), and average photosynthetic photon flux density (PPFD) of 600 μmol m^−2^ s^−1^ and were regularly irrigated. For stress treatment, we have divided greenhouse grown 1-month**-**old plants into two groups, one group for chilling stress and other as control group (unchilled plants). For chilling stress, ten plants per genotype were exposed to chilling temperature of 4 °C (day/night) in illuminated growth chamber for 5 days. For effective treatment, we have simultaneously engaged three chambers under same growth conditions, and samples were equally divided and placed at the center of each chamber. Samples were collected in triplicate from each genotype both from stress and control group and each parameter was analyzed as described below.

### Gas exchange and fluorescence measurements

Photosynthetic rate (A, μmol m^−2^ s^−1^), stomatal conductance (gs, mol H_2_O m^−2^ s^−1^), and transpiration rate (E, mmol m^−2^ s^−1^) were measured at morning time between 8:00 a.m. to 10:00 a.m. from fully expanded leaves (second from shoot apex) of each genotype (MT, *aur*, *hp1*). For gas exchange measurements, we have utilized a portable LI-6400XTR infrared gas analyzer (LI-COR Biosciences, USA) fixed at a constant chamber temperature of 25 °C with a LED light source to provide 600 μmol photons m^−2^ s^−1^. Stomatal aperture was measured as described in previous study [23].

Chlorophyll fluorescence parameters were measured on fully expanded leaves of the same plants that were selected for gas exchange analysis and data were analyzed on daily basis during entire treatment period by using portable fluorometer MINI-PAM (Walz, Effeltrich, Germany). Firstly, we have placed seedlings at dark for approx. 20 min followed by leaf surface exposure to a weak pulse of red light (0.03 μmol m^−2^ s^−1^) and the initial fluorescence (F_o_) was measured. Subsequently, leaves were exposed to 0.8 s of saturating actinic light (˃ 6000 μmol m^−2^ s^−1^) to measure maximum fluorescence (Fm). The potential quantum photochemical efficiency of PSII (Fv/Fm), operational photochemical efficiency of PSII (*Fq*′/*Fm*′), electron transport rate (ETR), and non-photochemical quenching coefficient (NPQ) were determined according to previous study [24].

### Determination of photosynthetic pigments and malondialdehyde content

Photosynthetic pigments (chlorophyll a, chlorophyll b, and carotenoids) in leaves were assayed through extraction by dimethyl sulfoxide (DMSO). The extraction sample was prepared (100 mg of fresh sample) in dark at the room temperature in acetone (80%), and absorbance readings were then determined with a UV/VIS spectrophotometer (Shimadzu UV-160, Kyoto, Japan).

The lipid peroxidation which is commonly measured in terms of malondialdehyde (MDA) content was analyzed by previously established method [24]. Briefly, 200 mg of leaves were collected from each genotype on the fifth day of the chilling treatment, followed by measuring the absorbance at 535 and 600 nm. Finally MDA content was calculated using an extinction coefficient of 155 mM^−1^ cm^−1^ and expressed as nmol g^−1^ FW.

### Determination of enzyme activities

For analyzing enzyme activities in control and stress plants, 350 mg of leaf samples was collected from each genotype and samples were then ground to powder using liquid nitrogen. A fresh mixture of 100 mM potassium phosphate buffer (pH 6.8), 0.1 mM EDTA, 100 mM phenylmethylsulfonyl fluoride, and 2 % (w/v) polyvinylpyrrolidone was prepared to homogenize the samples and later filtered through centrifugation at 13,000*g* for 20 min. The obtained supernatant was divided into small aliquots, and samples were stored at − 80 °C for enzymatic analyses. For catalase (CAT) enzyme activity, firstly, 1 mL reaction mixture was prepared that contained enzyme extract (25 μL), deionized water (350 μL), potassium phosphate buffer (50 mM) with adjusted pH 7, and hydrogen peroxide (15.625 mM; 30 % solution). For ascorbate peroxidase (APX) activity, we prepared a reaction mixture, which contained 2.9 mL of 100 mM sodium phosphate buffer with adjusted pH 7, 280 μL of deionized water, 0.1 mM EDTA-Na_2_, 0.3 mM ascorbic acid, and 0.06 mM H_2_O_2_. The SOD enzyme activity was performed based on the inhibition of the photoreduction of nitroblue tetrazolium. The activity of guaiacol peroxidase (POX) was measured as described previously [25]. Briefly, a total of 2 mL of a solution was prepared by adding 25 μl of the crude enzyme, 50 mM potassium phosphate buffer with adjusted pH 6.8, 20 mM guaiacol, and 20 mM of H_2_O_2_. Phenylalanine ammonium-lyase (PAL) was measured according to the previous method, which is based on rate of cinnamic acid production [26].

Next, we measured the activities of delta 1-pyrroline-5-carboxylate synthase (P5CS), pyrroline-5-carboxylate reductase (P5CR), ornithine aminotransferase (OAT), proline dehydrogenase (PDH), and proline oxidase (PO) according to methods described earlier [24, 27]. According to the quantification of the release of inorganic phosphorous, we have detected the activities for P5CS and P5CR. By calculating the extinction coefficient of 6220 M^−1^ cm^−1^ for NADH, we measured OAT activity. Based on reduction of NAD+ or FAD+, activities of PDH and PO were determined, respectively. Measurements of sucrose synthase (SS), sucrose phosphate synthetase (SPS), neutral and acid invertases (NI, AI), fructose 1,6-biphosphatase (FBPase), and fructokinase (FK) were performed as described previously [27]. Activity of BADH was detected based on its ability to reduce NAD+ to NADH, whereas activity of trehalose 6-phosphate synthase (T6PS) was quantified according to coupled assay.

### Determination of osmolytes concentration

The content of soluble sugars (glucose, sucrose, fructose and starch) was determined using previously described method [28]. Briefly, leaves were crushed to powder and 5 mL of aqueous methanol was added and samples were placed at room temperature in orbital shaker for overnight shaking. To precipitate the protein, we have well mixed the extract with 5 mL of perchloric acid (6% v/v) and centrifuge it at 6000*g* for 12 min. The clear supernatant was later used in the assay following the standard procedure. To measure starch concentration, we used enzymatic reaction as described previously [29]. The content of proline was detected as by freezing 0.5-g leaves and later homogenized in the presence of 5 mL of ethanol (96%), subsequently washed with 10 mL of 70% ethanol, and followed by centrifugation at 13,000*g* for 12 min. Afterwards, we collected 2 mL of supernatant and mixed it with 2.5 mL of glaciar acetic acid and 2.5 mL of ninhydrin reagent followed by incubation at 100 °C for approx. 45 min. Finally, 5 mL of benzene was added and absorbance was noted at 515 nm.

To estimate GB content, fresh extract from leaves were obtained in 2 NH_2_SO_4_ and frozen as described in previous method [30]. The extract was first mixed with triiodide solution (in equal amount), later vortexed and kept overnight at 4 °C. Following day, the mixture was centrifuged 12,000*g* at 4 °C for 20 min; subsequently, supernatant was discarded to get periodide crystals. Next, periodide crystals were dissolved in 10 mL of 1, 2-dichloroethane, vortexed, and kept at room temperature for approximately 20 min, followed by measuring the absorbance of solution at 365 nm. For choline measurement, we treated the samples with 0.5 units of Cho oxidase, 0.8 units of horseradish peroxidase, 118 μg of aminoantipyrine, and 40 μg of phenol in 200 μl of Tris–HCl (50 mM) at 37 °C for 30 min (pH 7.8). Choline was estimated by measuring the absorbance at 500 nm as described previously [24]. For measuring trehalose content, frozen leaves (0.2 g) were first homogenized at 4 °C after adding 2 mL of 20 mM phosphate buffer (PBS, pH 5.8) and then vibrated on a supersonic cell pulverizer (Ninbo Xin Yi Science Instrument Ltd., Co., Ninbo, Zhejiang) for 30 min. Absorbance of the samples was detected at 630 nm.

### Expression analysis

Total RNA was extracted with Trizol reagent (Invitrogen) and the first-strand cDNA was synthesized using 3 μg of RNA and 200 U of M-MLV reverse transcriptase (Invitrogen) according to the manufacturer’s protocol. RT-PCR was carried out to amplify fragments of *SlPHYA*, *SlPHYB1*, *SlPHYB2*, *SlPHYE*, and *SlPHYF* genes with 31 cycles using the first-strand cDNA as a template. In addition, actin was amplified with 24 cycles as an internal control. Real-time PCR was performed on an optical 96-well plate in an AB StepOnePlus PCR system (Applied Biosystems) by using SYBR Premix Reagent F-415 (Thermo Scientific). Relative gene expression was calculated using a relative quantification method [31]. All primers used in this study are listed in Supplementary Table S1.

### Statistical analysis

The experimental data were analyzed using two way ANOVA test, and significant statistical differences between means of treatment or genotype were measured by Tukey’s test at *P* < 0.05. qPCR was performed, and relative transcript expressions were measured by the 2^−ΔΔCt^ method. Heat map for osmoprotectants and enzymatic activities were created using R. Statistical analyses were performed using SPSS version 20 (SPSS Inc., USA).

## Results

### Impact of chilling stress on gas exchange parameters in tomato photomorphogenic mutants

**Fig. 1:**
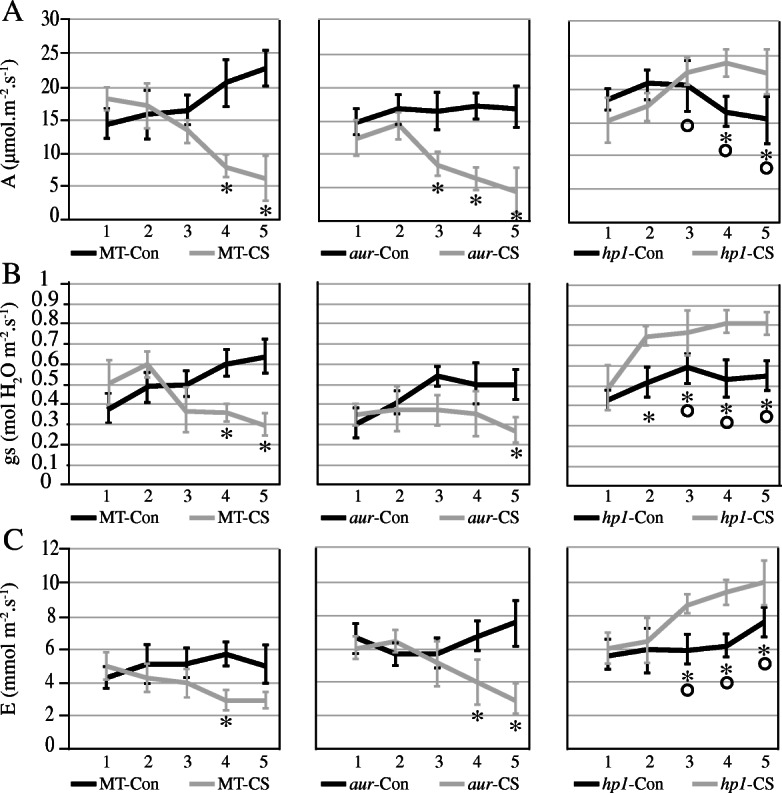
Effects of chilling stress on gas exchange parameters. **a** Photosynthetic rate denoted as A and measured in μmol m^−2^ s^−1^, **b** stomatal conductance denoted as gs and calculated in mol H_2_O m^−2^ s^−1^, and **c** transpiration rate denoted as E and calculated in mmol m^−2^ s^−1^. Samples were obtained from leaves of wild-type genotype (MT) and *aurea* (*aur*) and *high pigment1* (*hp1*) mutants of tomato cv. Micro-Tom. Blue lines indicate the effects on plants under control (Con) conditions whereas green lines show effects after chilling stress (CS). The data were collected and compared for each day until 5 days under chilling stress. Symbols that are not similar show significant differences (calculated by Tukey’s test, *P* < 0.05). Comparisons between the control and stress treatments within the same genotype were indicated as asterisks, whereas circles were used for comparisons among genotypes under the stress treatment

**Fig. 2:**
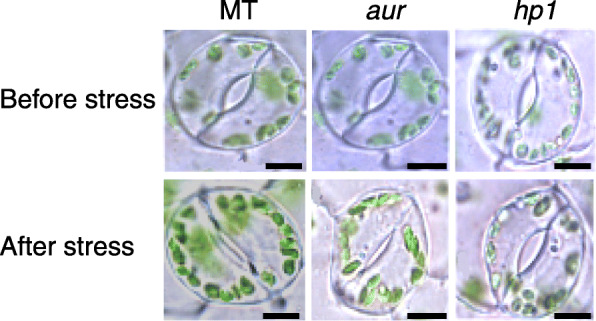
Effects of chilling stress on stomatal aperture of tomato photomorphogenic mutants. The changes in stomatal aperture of tomato *aur* and *hp1* mutants and the wild-type genotype (MT) were observed before and after chilling stress. The lower surfaces of the leaves with or without stress were examined under a microscope

Both temperature and light are important environmental factors that can directly affect plant growth and development in any geographical location. Photosynthesis is probably the first event which can be seriously altered by temperature and light fluctuations during a complex network of signaling pathways. In this study, we found that photosynthetic rate, stomatal conductance, and transpiration rate were significantly increased in tomato phytochrome-sensitive *hp1* mutant upon subjecting to chilling stress (Fig. 1). However, starting from third day of chilling stress, we observed a decreasing trend in these parameters both in MT and phytochrome-deficient *aur* mutant (Fig. 1). Intriguingly, phytochrome-sensitive mutant *hp1* that shows higher transpiration rate after third day compared to control and thus coincides with its increased values for stomatal conductance. This finding directly indicates that photosynthetic rate was efficiently maintained in *hp1* during chilling stress (Fig. 1). This idea was further validated through stomatal closure examination. The confocal microscope examination indicated no significant differences in pore width between the wild-type MT and both *aur* and *hp1* mutants under control condition (Fig. 2). Importantly, under chilling stress, a noticeable shrinkage in the pore width was recorded in MT and *aur* (Fig. 2). In contrast, the stomatal closure of *hp1* mutant did not change and the pore width remained constant under chilling stress (Fig. 2).

### Effect of chilling stress on chlorophyll fluorescence in phytochrome mutants of tomato

**Fig. 3:**
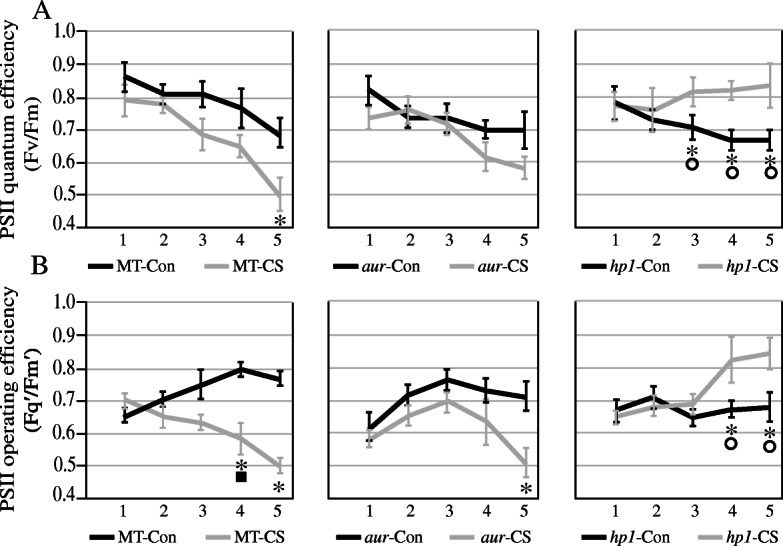
Comparison of **a** PSII potential quantum efficiency (Fv/Fm) and **b** PSII operating efficiency (Fq′/Fm′) in leaves of the wild-type genotype (MT) and *aurea* (*au*) and *high pigment1* (*hp1*) mutants of tomato cv. Micro-Tom under control (Con) and chilling stress (CS) for 5 days. Blue lines represent data for control and green lines show data for stress treatment. Symbols that are not similar show significant differences (calculated by Tukey’s test, *P* < 0.05). Comparisons between the control and stress treatments within the same genotype were indicated as asterisks, whereas solid squares represent comparisons among genotypes under the control treatment and circles were used for comparisons among genotypes under the stress treatment

**Fig. 4:**
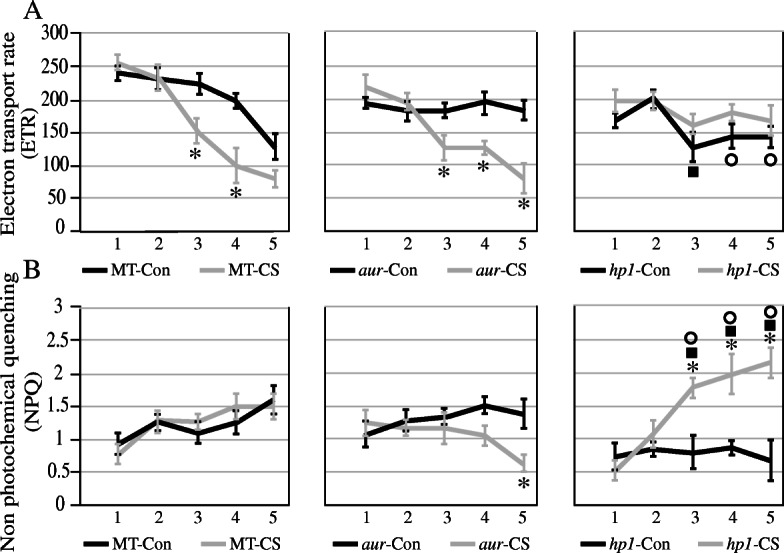
Effect of chilling stress on **a** electron transport rate (ETR) and **b** non-photochemical quenching (NPQ) in all three tomato genotypes under control (Con) and chilling stress (CS). Blue lines represent data for control and green lines show data for stress treatment. Symbols that are not similar show significant differences (calculated by Tukey’s test, *P* < 0.05). Comparisons between the control and stress treatments within the same genotype were indicated as asterisks, whereas solid squares represent comparisons among genotypes under the control treatment and circles were used for comparisons among genotypes under the stress treatment

Measurement of chlorophyll fluorescence parameters is crucial for characterizing the photosynthetic activity in plants. In this study, we have analyzed that potential quantum efficiency of PSII (Fv/Fm value) and PSII operating efficiency (*Fq*′/*Fm*′ value) were decreased significantly both in MT and *aur* mutant (Fig. 3a and b, respectively). Moreover, electron transport rate (ETR) was also significantly decreased in MT and *aur* mutant starting from the third day of chilling stress (Fig. 4a), whereas non-photochemical quenching (NPQ) was not affected in MT but declined in *aur* mutant after chilling stress (Fig. 4b). In case of *hp1* mutant, we found that PSII quantum efficiency, PSII operating efficiency, and NPQ values were increased significantly under chilling stress; however, ETR value was not much affected during stress (Figs. 3 and 4). The result indicates that *hp1* mutant showed enhanced photosynthetic performance under chilling stress as compared to MT and *aur* mutant. A significant increase of NPQ in *hp1* mutant specifically after third day of stress treatment indicate that more light energy is absorbed and dissipated through non-photochemical pathways in order to enhance photosynthetic efficiency of PSII as correlated with its high Fv/Fm value under the chilling stress.

### Chilling stress and its effect on photosynthetic pigments in two phytochrome mutants of tomato

Chlorophyll content depends on physiological state of a plant, and it is a strong indicator of stress tolerance in tomato [32]. In this work, we have found that mutant *aur* had the lowest content of chlorophyll a, chlorophyll b, and carotenoids than MT and *hp1* mutant both with and without stress (Table 1). Interestingly, *hp1* mutant showed higher contents of chlorophyll a and carotenoids; however, decrease in chlorophyll b content and chlorophyll a:b ratio was observed after chilling stress (Table 1). In case of MT, chlorophyll a, chlorophyll b, and chlorophyll a:b ratio were all decreased, but slight increase in carotenoids was noticed after chilling stress (Table 1).

**Table 1:** Comparison of photosynthetic-related pigments in wild-type and two phytochrome mutants of tomato under chilling stress

Treatment	Genotypes	Chlorophyll a	Chlorophyll b	Chlorophyll a:b	Carotenoids
		μg cm^−2^	μg cm^−2^		μg cm^−2^
Control	MT	22.37 ± 1.02 ^Aab^	10.04 ± 1.12 ^Aab^	4.2 ± 0.12 ^Aab^	5.2 ± 0.83 ^Ab^
	*aur*	17.21 ± 1.21 ^Aa^	8.33 ± 0.72 ^Aa^	3.3 ± 0.24 ^Aa^	3.4 ± 0.33 ^Aa^
	*hp1*	25.09 ± 0.75 ^Ab^	14.09 ± 0.42 ^Ab^	4.9 ± 0.37 ^Ab^	7.5 ± 0.28 ^Ac^
Chilling stress	MT	19.71 ± 1.02 ^Aa^	7.34 ± 0.72 ^Aab^	3.8 ± 0.38 ^Ab^	6.1 ± 0.22 ^Ab^
	*aur*	16.34 ± 1.05 ^Aa^	5.28 ± 0.52 ^Aa^	2.8 ± 0.61 ^Aa^	2.5 ± 0.64 ^Aa^
	*hp1*	32.52 ± 1.25 ^Bb^	9.79 ± 0.44 ^Ab^	3.5 ± 0.82 ^Aab^	13.2 ± 0.62 ^Bc^

Different letters indicate significant differences (Tukey’s test, *P* < 0.05). Upper case letters indicate comparisons between control and stress within the same genotype. Lower case letters indicate comparisons among genotypes under the same treatment (control or stress)

### Impact of chilling stress on antioxidant enzymes’ activity and MDA content in tomato photomorphogenic mutants

**Fig. 5:**
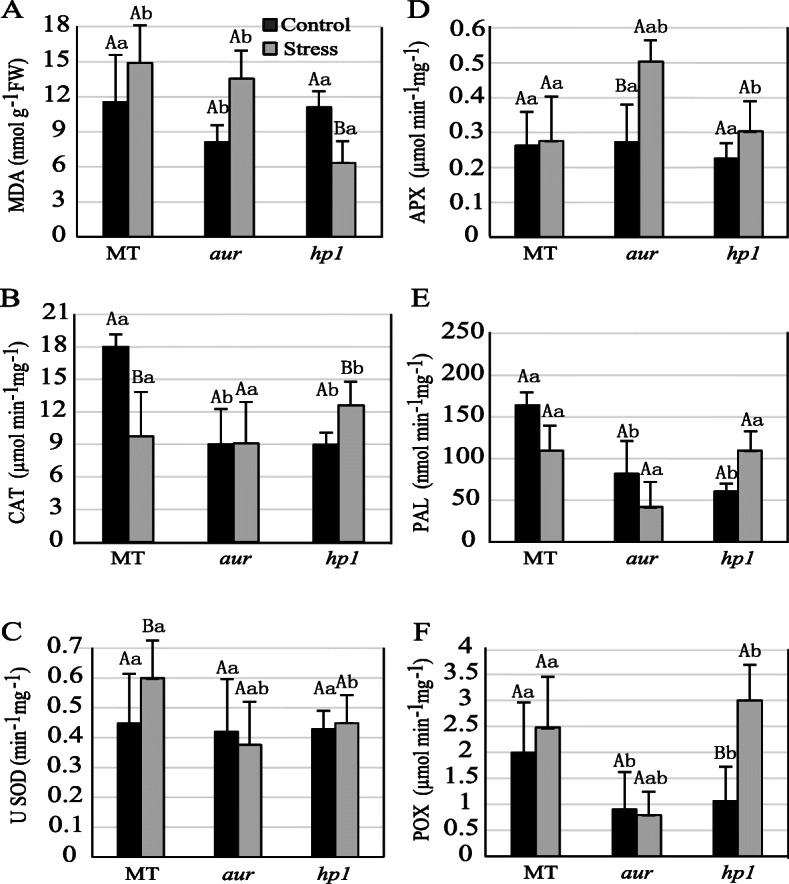
Effect of chilling stress on **a** malondialdehyde (MDA) content and **b**–**f** activities of antioxidant enzymes in leaves of the wild-type genotype (MT) and *aurea* (*aur*) and *high pigment1* (*hp1*) mutants of tomato cv. Micro-Tom under control and chilling stress conditions. Symbols that are not similar show significant differences (calculated by Tukey’s test, *P* < 0.05). Comparisons between control and stress within the same genotype were denoted in upper case letters, while comparisons among genotypes under the same treatment (control or stress) were denoted in lower case letters

In this study, we have analyzed MDA contents and activities of antioxidant enzymes in all tomato genotypes before and after chilling stress. A chilling-induced injury exacerbates ROS production in plant’s cell which cause oxidative stress and culminate in severe damages at the cellular level. It directly or indirectly leads to cellular membrane damage, protective enzymes’ deactivation, and ionic imbalances in plants. Lipid peroxidation is a process of degradation of lipids by free radicals and is commonly measured by overproduction of MDA contents. Interestingly, we have found that MDA levels were increased in MT and *aur* mutant, whereas in *hp1* mutant its level was decreased significantly under chilling stress (Fig. 5a). Moreover in *hp1* mutant under chilling stress, we have found marked increase in the activities of antioxidant enzymes including APX, CAT, PAL, and POX, while activity of SOD was not affected that much as compared to control (Fig. 5b–f). In case of *aur* mutant, enzyme activities were reduced under chilling stress except for APX (Fig. 5b–f). For MT, we have observed perplexed phenomena, where activities for CAT and PAL decreased but enzyme activities for POX and SOD were increased while no significant effect on APX activity was noticed after chilling stress (Fig. 5b–f).

### Impact of chilling stress on osmoprotectant synthesis and accumulation in tomato photomorphogenic mutants

**Fig. 6:**
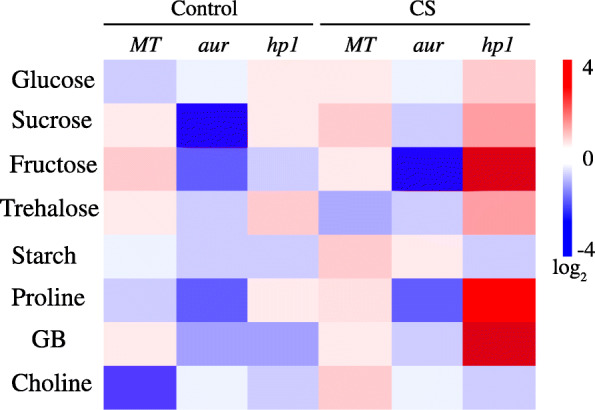
Heat map of selected set of stress-related compounds in leaves from wild-type genotype (MT) and *aurea* (*aur*) and *high pigment1* (*hp1*) mutants of tomato under control and chilling stress (CS) conditions. Red color represents higher relative concentration and blue color represents lower relative concentration. Scale is the log_2_ of the mean concentration values after normalization (*n* = 10). The data is representative of two independent experiments

**Fig. 7:**
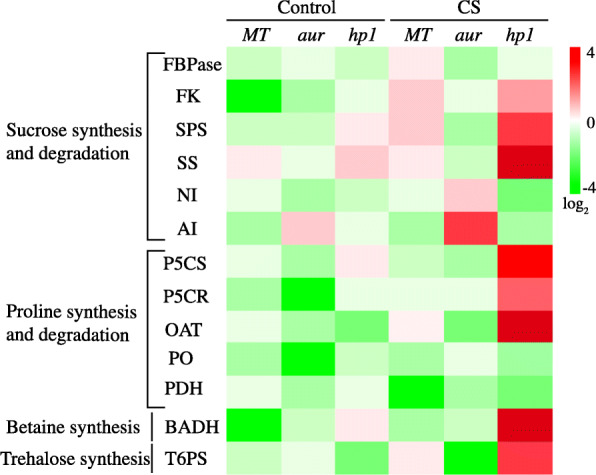
Heat map of a selected set of enzymes related to the different osmolytes biosynthetic pathways (synthesis and degradation). The enzyme activities were measured in in leaves from wild-type genotype (MT) and *aurea* (*aur*) and *high pigment1* (*hp1*) mutants of tomato under control and chilling stress (CS) conditions. Red color represents higher relative activity and yellow color represents lower relative activity. Scale is the log_2_ of the mean concentration values after normalization (*n* = 10). The data is representative of two independent experiments

Osmoprotectants are also called as compatible solutes which accumulate in plants when exposed to stress conditions. When plants are exposed to any stress, plants tend to stabilize proteins and membranes as first response to avoid damages by osmotic pressure, thereby accumulating higher amounts of different osmoprotectants [33]. In this study, we have comprehensively analyzed some of the most important osmoprotectants in plants that include soluble sugars, quaternary ammonium compounds, trehalose, and proline under chilling stress and control conditions in tomato genotypes in order to evaluate the regulation of these compounds. In addition, we have also attempted to explore activities of selected group of enzymes in the biosynthetic pathway of each osmoprotectants to investigate how their accumulation is controlled at the molecular or biochemical level in MT and phytochrome mutants under chilling stress. The results for osmoprotectants’ accumulation and associated enzyme activities were represented as log_2_ heat map of the absolute values obtained for three genotypes both under control and stress conditions (Figs. 6 and 7, respectively).

The concentrations of soluble sugars, starch, trehalose, choline, and glycine betaine (GB) under control and chilling stress conditions are represented in Fig. 6. Interestingly, we have found an obviously different accumulation pattern for these compounds in *hp1* mutant as compared to MT and *aur* under stress. Under chilling stress, a dramatic accumulation of fructose was detected in *hp1* mutant among other soluble sugars; overall, soluble sugars were increased in *hp1* under stress as compared to MT and *aur* mutant. For starch, we have found its slight accumulation both in MT and *aur* while no difference was detected in *hp1* after stress (Fig. 6). In case of proline, we have found the lowest accumulation in *aur* mutant while in *hp1* its accumulation was fourfold higher as compared to control (Fig. 6). Under control condition, there was no difference between *aur* mutant and *hp1* for GB accumulation; however, its accumulation was approximately eightfold higher in *hp1* under chilling stress as compared to control. For choline, we have noticed its considerable accumulation only in MT under stress while no difference was noticed in both mutants before and after chilling stress (Fig. 6).

In order to further deepen our understanding, next we have analyzed the activities for main enzymes in biosynthetic pathway (including synthesis and degradation) of selected osmoprotectants, i.e., sucrose, proline, GB, and trehalose, results of which are represented in Fig. 7. As expected, we have noticed different activity pattern in three tomato genotypes under chilling stress. In case of sucrose accumulation in *hp1* mutant, we have observed enhanced activities of enzymes involved in synthesis of sucrose (FK, SS, SPS), while enzymes involved in its degradation (NI, AI) were inhibited under chilling stress (Fig. 7). Similarly, for MT, we have observed only slight increase in the activities of sucrose synthesis enzymes but inhibition of sucrose degradation enzymes. In case of *aur* mutant under chilling stress, we have observed increased activities only for sucrose inhibiting enzymes while activities for sucrose synthesis enzymes were reduced (Fig. 7). For proline, no comparable differences were found in the activities for proline synthesis and degrading enzymes both in MT and *aur* mutant. In contrast, a sharp increase in the activities of proline synthesis enzymes (OAT, P5CR, P5CS) but reduced activities for proline degrading enzymes (PDH, PO) were noticed in *hp1* mutant after chilling stress (Fig. 7). As for GB, we have noticed eightfold increase for the activity of BADH, the rate limiting enzyme for GB, in *hp1* mutant under stress as compared to control. Similarly, activity of T6PS (rate limiting enzyme for trehalose synthesis) was strongly induced in *hp1* after stress. On the other hand, not any notable differences were observed for the activities of BADH and T6PS in *aur* mutant before and after stress. However, activity of T6PS was slightly increased in MT after chilling stress as compared to control (Fig. 7).

### Impact of chilling stress on transcript levels of phytochrome genes (PHYs) in tomato photomorphogenic mutants

**Fig. 8:**
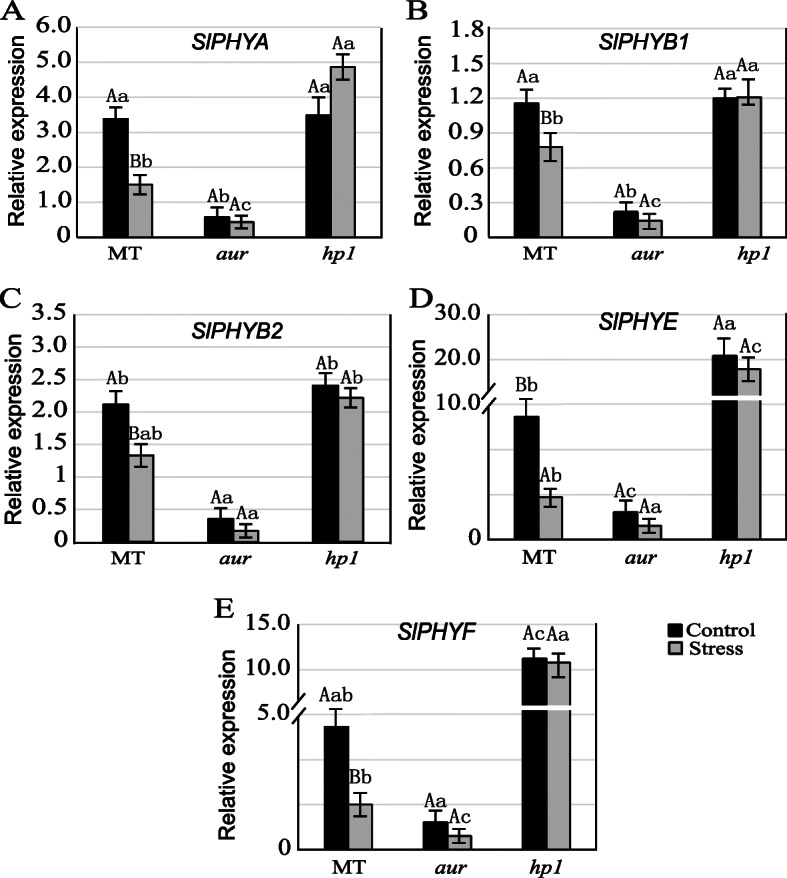
Analysis of PHY genes in tomato mutants, *aurea* (*aur*) and *high pigment1* (*hp1*), and wild-type tomato (MT) under control and CS conditions. Transcript levels of all five known tomato PHY genes **a** *SlPHYA*, **b** *SlPHYB1*, **c** *SlPHYB2*, **d** *SlPHYE*, and **e** *SlPHYF* were examined in mutants and wild-type genotypes before and after chilling stress

Recently, phytochromes (PHYs) were reported to act as molecular switches in response to several abiotic stresses [5, 34]. For further exploration of the effect of chilling stress on phytochromes at the molecular level, expression of the known five tomato phytochrome genes *SlPHYA*, *SlPHYB1*, *SlPHYB2*, *SlPHYE*, and *SlPHYF* were analyzed. The results showed a significant induction in transcript levels of *SlPHYA*, *SlPHYB1*, *SlPHYB2*, *SlPHYE*, and *SlPHYF* genes in *hp1* mutant under control and chilling stress conditions compared to those of MT (Fig. 8). Conversely, a marked reduction in the transcript levels of these genes were recorded in *aur* mutant under control and chilling stress conditions (Fig. 8). Notably, the induction levels of *SlPHYE* and *SlPHYF* in *hp1* mutant were strikingly higher than other PHY genes after exposure to stress (Fig. 8). These findings suggest that light induction and signal transduction in *hp1* mutant become more active under stress conditions as compared to wild type. As for *aur* mutant, a reverse phenomenon was observed and expressions of all PHY genes were repressed during chilling stress (Fig. 8).

## Discussions

Phytochromes are photoreceptors that absorb red and far-red light and therefore play integrating roles between light signaling, environmental conditions, and plant development [35]. In plants, phytochromes are encoded by small gene families; for example, there are five phytochrome members, *phy A* to *phy E*, in Arabidopsis and tomato, whereas monocotyledonous rice has three members, i.e., phy A to phy C [36]. Phytochromes have been shown to be involved in several photomorphogenic responses, for example, photocontrol of seed germination, plant architecture, biomass accumulation, stem elongation, leaf development, and flowering [35, 37]. More recently, it has been well established that phytochromes play vital roles in number of different abiotic stresses such as water stress and are crucial part of signaling pathways in plants [5, 38]. To establish the link between phytochromes and stress factors, numerous studies have employed phytochrome mutants to evaluate phytochrome-mediated responses in plants. For example, phytochrome B mutants (*phyB*) of rice [39] and arabidopsis [40] show enhanced cold tolerance as compared to wild-type, attributed to a lower electrolyte leakage and MDA content than in WT, thus maintaining the cell membrane integrity in *phyB* mutants. Another study further revealed the interaction of PIF family gene (*OsPIL16*) with rice phyB, which is necessary to regulate *OsDREB1* expression and therefore enhanced the membrane integrity and contributed positively for cold tolerance in *phyB* mutants. In another study, it was revealed that *phyA* and *phyB* function in an opposite manner to regulate cold tolerance in tomato [20]. In the same study, WT and five phytochrome mutants, *phyA*, *phyB1*, *phyB2*, *phyB1B2*, and *phyAB1B2*, were used and their responses were compared under cold tolerance exposed to dark, red (R), and far-red light (FR) conditions. Surprisingly, the cold tolerance was increased in WT subjected to FR as demonstrated by changes in Fv/Fm, survival rate, and relative electrolyte leakage. Under FR conditions, there was no difference in cold tolerance for *phyA* and *phyB1B2*; however, *phyA* had decreased cold tolerance under R conditions. Additionally, the cold tolerance for *phyB1*, *phyB2*, and *phyB1B2* mutants were increased under FR. The authors thus concluded that *phyA* and *phyB* serve antagonistic functions to cold response under FR. Further, FR activates *phyA* to induce ABA and JA signaling, which in turn triggers CBF pathway genes to positively regulate cold tolerance in tomato [38]. Another interesting report unveiled that the accumulation of anthocyanin in Arabidopsis is promoted by JA under FR and is dependent on *phyA* [41]. All these studies have revealed a strong relationship of phytochromes with abiotic stress signaling network in plants, thereby affecting several physiological, biochemical, and molecular parameters.

In world ranking, tomato is second most important horticultural crop; however, cultivated tomato species suffers greatly from chilling temperatures (1–10 °C) and fails to acclimate to low temperature [18, 42]. Chilling temperature is a major environmental threat that seriously affects tomato growth and development and influences its productivity. Unlike previous studies where mutants belong to specific phytochrome family, in this study, we utilized two phytochrome mutants in tomato, phytochrome-deficient mutant *aur* and phytochrome-sensitive mutant *hp1*, including wild-type tomato cv. Micro-Tom. We evaluated these tomato genotypes and compared their physiological, biochemical, and molecular parameters under chilling stress. Intriguingly, we found that phytochrome-sensitive mutant *hp1* respond differently under chilling stress than MT and phytochrome-deficient *aur* mutant, although under control conditions we did not find such variations in most of the parameters studied here. The gas exchange parameters including photosynthetic rate, stomatal conductance, stomatal aperture, and transpiration rate, all were significantly reduced in MT and *aur* mutant, but enhanced in *hp1* mutant under chilling stress (Figs. 1 and 2). In addition, fluorescence parameters showed improved photosynthetic efficiency in *hp1* under chilling stress as compared to MT and *aur* mutants (Figs. 3 and 4). This apparent tolerance in *hp1* mutant can be associated with increased activities of antioxidant enzymes (Fig. 5) and higher accumulation of osmoprotectants than MT and *aur* mutant plants (Figs. 6 and 7). The following discussion investigates the differences of physiological, biochemical, and molecular parameters among tomato genotypes in details, which would elaborate the underlying molecular mechanism related to chilling stress.

As we found an obvious reduction in photosynthetic rate, stomatal conductance transpiration rate, along with some changes in fluorescence related parameters such as chlorophyll contents, in MT genotype under chilling stress, which support that the treatment has produced effective stress conditions. Previous reports have demonstrated such changes in physiological parameters under induced stress conditions in many plant species [43, 44]. Under chilling stress, stomatal conductance, stomatal aperture, and transpiration rate were decreased in MT and *aur* mutant, which has been reflected by reduced photosynthetic rate in both genotypes (Figs. 1, 2, 3, and 4). Low temperature can severely affect photosynthesis by disturbing the balance between RuBP regeneration and carboxylation; consequently, the diffusion of CO_2_ becomes limited that restrains the RUBISCO activity resulting in decreased photosynthetic rate. In other studies, a similar reduction in gas exchange parameters in different plant species was observed during low temperatures [45, 46]. On the other hand, we have noticed enhanced performance of gas exchange parameters including stomatal conductance, stomatal aperture, transpiration rate, and photosynthetic rate after chilling stress in *hp1* plants (Figs. 1, 2, 3, and 4), which can be specifically related to significant increase of NPQ after stress (Fig. 4). Because of the fact that *hp1*, a high pigment tomato mutant, which is characterized by its exaggerated response to light and most likely saturate photosynthetic rate in the leaves. This could possibly saturate excitation energy inside mesophyll cells of leaves which then cause photoinhibition of PSII and result in a decrease in quantum yield and photosynthetic rate [47]. Interestingly, plants have developed several protective mechanisms to avoid photo-damage, including non-photochemical quenching (NPQ). NPQ protects the photosynthetic apparatus by quenching excitation energy of PSII antennae and converts this energy into thermal energy which later release in the form of heat [48]. After third day of chilling stress, we have observed a significant increase of NPQ in *hp1* plants (Fig. 4); this directly exerts an effect on the rate of PSII photochemistry (Fig. 3) and releases chlorophyll excitation energy as heat within PSII, which is directly associated with preventing the onset of photoinhibition [49]. Notably, we did not observe any difference of NPQ before and after chilling stress in MT; however, it was slightly decreased after chilling stress in *aur* mutant (Fig. 4), which implies that *aur* plants are more sensitive to photoinhibition. It is well established that Fv/Fm ratio represents the maximum quantum efficiency of PSII photochemistry [50]. This parameter has been measured and commonly used to detect imbalance in the photosynthetic apparatus induced by stress, since photo-damage to the reaction centers can decrease the values of Fv/Fm [51]. As expected, Fv/Fm values were decreased after chilling stress both in MT and *aur* but increased in *hp1* from third day onwards (Fig. 3), which are consistent with our results for NPQ and further confirm preventing the photoinhibition in *hp1* plants. In addition, both in MT and *aur* mutant, PSII operating efficiency and electron transport rate were reduced (Figs. 3 and 4). According to these findings, we can deduce that stomatal limitations during photosynthesis lessen the depletion of ATP and NADPH, which consequently impaired the electron transport rate and reduced the value of Fq′/Fm′ as described in previous reports [51, 52].

Generally, membrane lipid peroxidation in plants is measured in terms of MDA and is considered a reliable marker for oxidative stress. Intriguingly, MT and *aur* plants had higher MDA contents under chilling stress (Fig. 5a), which implies elevated lipid peroxidation and represents excessive damage to the membranes due to ROS activity. Consistently, MT and *aur* display low PSII operating efficiency (Fig. 3), and low ETR (Fig. 4) under stress. As expected, a low level of MDA was noticed for *hp1* mutant (Fig. 5a), which specify that *hp1* has greater ability to tolerate oxidative damages under chilling conditions. These results were also confirmed from the activities for antioxidant enzymes in *hp1*, which were mostly triggered after chilling stress (Fig. 5). A large body of evidence indicates that when plants are exposed to any environmental stress, it could perturb oxidative balance leading to increase MDA accumulation due to overproduction of ROS. This leads to disruptions in many vital processes in plants including plant metabolism and photosynthesis efficiency; however, plants do have a sophisticated innate mechanism to regulate the excessive production of ROS by regulating the antioxidant enzymes [53, 54].

A very effective way of responding to stress factors and to mitigate osmotic pressure in plants is to synthesize and accumulate osmoprotectants [33]. To divulge more into molecular mechanisms of stress responses of tomato genotypes in this study, we have analyzed endogenous accumulation of some of the most important osmoprotectants (such as proline, quaternary ammonium compounds, soluble sugars, and trehalose; Fig. 6) and activities of their biosynthesis related enzymes (Fig. 7) before and after chilling stress for each genotype, and results are compared and represented as a log_2_ heat map. Under chilling stress, an obvious difference was found for the accumulation of osmoprotectants in *hp1* mutant than both for MT and *aur*. For instance, sugars (glucose, sucrose, fructose), which are considered as typical osmoprotectants, were accumulated in higher amounts in *hp1* after chilling stress (Fig. 6). Previously, it has been validated that the monosaccharide sugars (glucose and fructose) and disaccharides (sucrose, trehalose), which are typical osmoprotectants, serve as cellular membrane stabilizer, ROS scavengers, and signaling molecules and increase plant tolerance to chilling and freezing stress [55]. On contrary, we did not observe any significant difference of sugar accumulation before and after stress in *aur* mutant (Fig. 6). While in MT, no difference was observed for sucrose and fructose accumulation before and after stress, however, glucose and trehalose were significantly increased and decreased, respectively, after chilling stress (Fig. 6). Moreover, we noticed a dramatic increase in GB and proline content in *hp1* (Fig. 6). Proline and GB are plant metabolites that safeguard proteins and other cell membranes under stress to conserve cellular functions [56]. The accumulation of proline was essentially carried out through activities of its biosynthesis enzymes P5CS, P5CR, and OAT (Fig. 7). It is also evident that GB stabilizes the efficiency of PSII photochemistry, thereby stopping the dissociation of regulatory proteins from the core complex. Notably, it has been previously established that hyperactive mutant plants overaccumulated several metabolites; few of them were involved in antioxidant activities [57]. Therefore, our results are consistent in light of these studies and confirm that *hp1* plants have the ability to overcome oxidative stress under chilling temperature as compared to phytochrome-deficient *aur* mutant as well as MT.

**Fig. 9:**
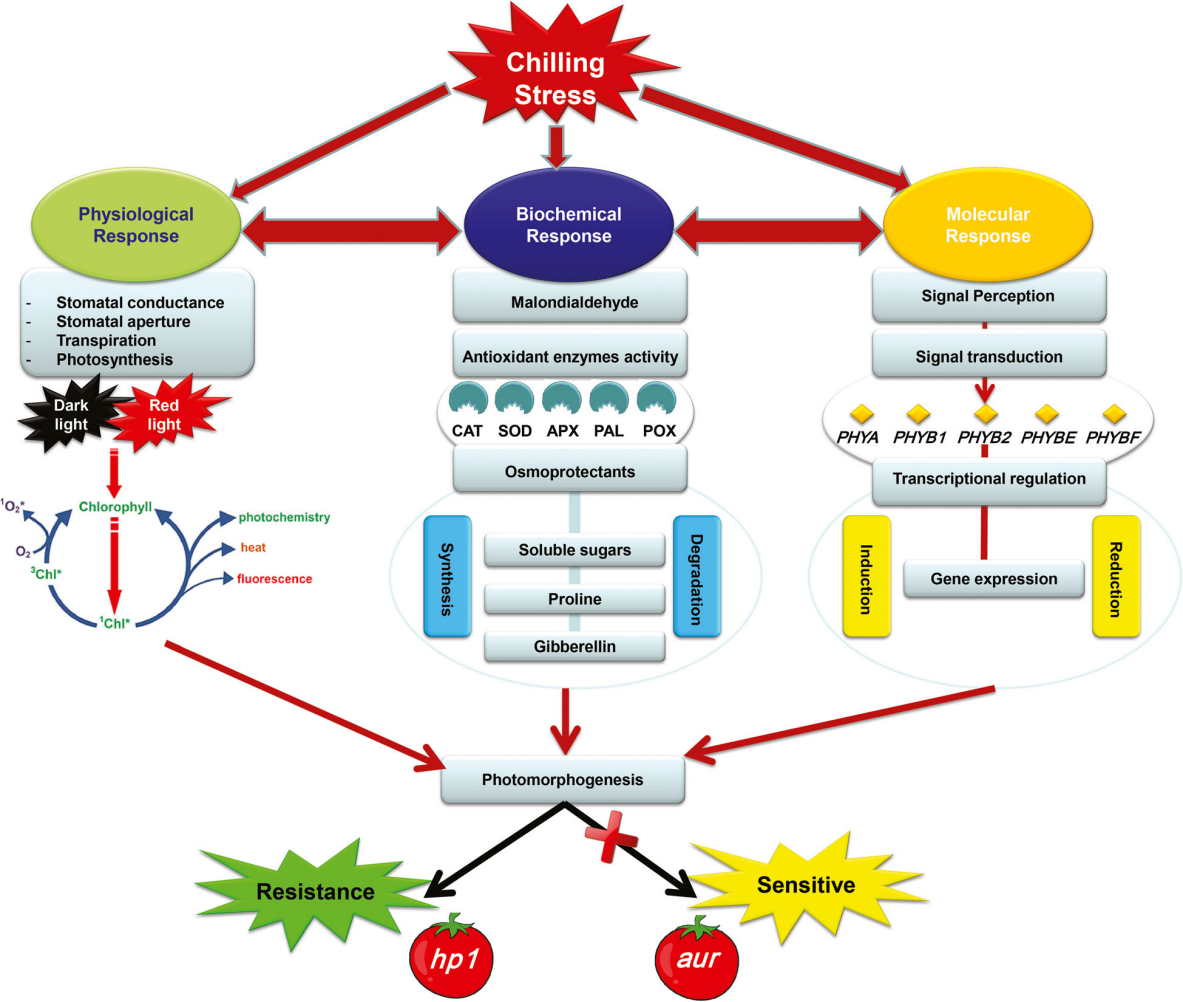
A schematic diagram showing divergent regulatory mechanisms of phytochrome-sensitive and phytochrome-deficient tomato mutants in response to chilling stress

At molecular level, PHY genes are thought to be activators of various mechanisms that merge diverse responses during chilling acclimation; hence, plants are considerably able to improve their tolerance to cold stress after subjection to non-chilling temperatures [57, 58]. This hypothesis can construe the higher transcript levels of PHY genes in the *hp1* mutant than in both *aur* mutant and the wild-type MT under chilling stress condition (Fig. 8). This study identified that *hp1* mutant which is hyper responsive to light show better photosynthetic performance under chilling stress by stabilizing PSII photochemistry and increasing photosynthetic rate. However, phytochrome-deficient mutant *aur* and wild-type MT plants show a different pattern, where most physiological parameters were negatively affected under chilling stress. Several studies show that chilling stress reduce the ability of ROS scavenging enzymes which ultimately results in photoinhibition and abnormal photosynthesis. Interestingly, we have found that this increased tolerance in *hp1* under chilling stress could be attributed to their ability to reduce the oxidative damage. Moreover, overaccumulation of several osmolytes, higher activities of antioxidant enzymes, and upregulation of PHYs also played crucial role for tolerance mechanisms in *hp1* genotype as illustrated in our schematic diagram (Fig. 9). On contrary, *aur* mutant did not accumulate significant amount of osmolytes and activities of most antioxidant enzymes were reduced under chilling stress, which could clearly explain the mechanism of sensitivity to chilling stress in this genotype (Fig. 9).

## Conclusions

This study focuses on two contrasting phytochrome mutants of tomato namely *aur* and *hp1* in order to understand phytochrome-mediated regulation of stress responses in tomato. Our results show number of physiological, biochemical, and molecular aspects that are positively regulated in *hp1* genotype; on the other hand, similar parameters are negatively regulated in *aur* and wild-type genotypes under chilling stress. Overall, our findings show enhanced tolerance of *hp1* genotype under chilling stress mainly due to reduced oxidative damages through activation of various protective mechanisms. In a nutshell, this work thus establishes a significant link between phytochromes and their role in stress tolerance and could be useful for future research.

## Supplementary Information

**Additional file 1:** **Supplementary Table S1.** Primers list used for qRT-PCR based tomato PHY genes expression.

## Data Availability

All the data required for the processing of the conclusions are presented in the “Results” section.

## Abbreviations

*aur*: *Aur*ea phytochrome-deficient mutant
*hp1*: *High pigment1* phytochrome-sensitive mutant
MT: Micro-Tom wild type tomato cultivar
NPQ: Non-photochemical quenching
PPFD: Photosynthetic photon flux density
MS: Murashige-Skoog
Fm: Maximum fluorescence
DMSO: Dimethyl sulfoxide
MDA: Malondialdehyde
CAT: Catalase
APX: Ascorbate peroxidase
SOD: Superoxide dismutase
POX: Peroxidase
PAL: Phenylalanine ammonium-lyase
GB: Glycine betaine
P5CS: Delta 1-Pyrroline-5-carboxylate synthase
P5CR: Pyrroline-5-carboxylate reductase
OAT: Ornithine aminotransferase
PDH: Proline dehydrogenase
PO: Proline oxidase
SS: Sucrose synthase
SPS: Sucrose phosphate synthetase
NI: Neutral invertases
AI: Acid invertases
FBPase: Fructose 1,6-biphosphatase
FK: Fructokinase
T6PS: Trehalose 6-phosphate synthase
