# Supplementary material for: Comparative analysis of two phytochrome mutants of tomato (Micro-Tom cv.) reveals specific physiological, biochemical, and molecular responses under chilling stress
Source: J Genet Eng Biotechnol. 2020 Nov 27;18:77. doi: 10.1186/s43141-020-00091-1 (PMC7695757; doi:10.1186/s43141-020-00091-1)
Supplement: Supplementary file 1 — Additional file 1: Supplementary Table S1. Primers list used for qRT-PCR based tomato PHY genes expression. [file 43141_2020_91_MOESM1_ESM.docx]

| Gene | Primer for qRT-PCR | Locus |
| --- | --- | --- |
| Sl-PHYA | F: 5´ - CACTCTCGTGGAGGATTCAT - 3´/ R: 5´ - GAGCCATAAAACACACACCC - 3´ | Solyc10g044670 |
| Sl-PHYB1 | F: 5´ - ACTTCTGTTCGGTCCATTCC - 3´ / R: 5´ - TCTCAGACAACTGTGATGCC - 3´ | Solyc01g059870 |
| Sl-PHYB2 | F: 5´ - GTGAGGGTTATTCAGGATGA - 3´ / R: 5´ - TGACCATATACTGAGGGTGAC - 3´ | Solyc05g053410 |
| Sl-PHYE | F: 5´ - CGCTATTGAGGAACCCACTT - 3´ / R: 5´ - GCATCAACACCAATCAGACC - 3´ | Solyc02g071260 |
| Sl-PHYF | F: 5´ - ACTAGCCAAGATCATTGACG - 3´/ R: 5´ - CTCCAAGATTGAACTCACAAG - 3´ | Solyc07g04548 |

Supplementary Table S1: Primers list used for qRT-PCR based tomato PHY genes expression.
